# Advances in the Regulation by Immune Cells of Skeletal Myositis Outcomes

**DOI:** 10.14336/AD.2025.0549

**Published:** 2026-07-14

**Authors:** Shuxian Huang, Yongling Zhao, Yuanpeng Xin, Ziqi Yao, Yi Deng, Qihui Cai, Junyi Xie, Ruixue Wang, Zhaohong Liao

**Affiliations:** ^1^Department of Laboratory Medicine, School of Medicine, Foshan University, Foshan, 528000, China.; ^2^Guangxi Key Laboratory of Tumor Immunology and Microenvironmental Regulation, Guilin Medical University, Guilin, Guangxi, 541199, China.; ^3^School of Medicine, Xiamen University, Xiamen, Fujian, 361102, China.; ^4^Department of Pathogen Biology, Shenzhen University Medical School, Shenzhen, Guangdong, 518052, China.; ^5^Departments of Basic Medicine, School of Medicine, Foshan University, Foshan, 528000, China.; ^6^Guangdong Provincial Key Laboratory of Construction and Detection in Tissue Engineering, Department of Anatomy, School of Basic Medical Sciences, Southern Medical University, Guangzhou, 510515, China.

**Keywords:** skeletal myositis, organ failure, immune cells, cytokines, antibodies, clinical management

## Abstract

Skeletal myositis prevalence is increasing annually, impairing mobility and potentially causing acute organ failure. Following muscle injury, various factors within damaged muscle trigger immune cell exudation from the bloodstream to the injured area, influencing disease progression. This paper synthesizes global research on cellular/molecular mechanisms in skeletal myositis, examining immune cell regulation in exogenous-induced skeletal myositis, dystrophic myopathy, and idiopathic inflammatory myopathy (IIM). We conclude that cytokines from intramuscular immune cells represent key targets for clinical management and therapeutic development.

## 1. Introduction

Skeletal muscle constitutes approximately 40% of body weight and is essential for generating movement, maintaining posture, maintaining internal structural stability, thermoregulation, and facilitating blood and lymphatic circulation [1]. It also plays a crucial role in maintaining metabolic processes and vital signaling homeostasis [2]. Muscle function dynamically responds to aging, genetics, disease, pharmacological interventions, and mechanical injury. Microscopic regulation involves signaling pathway (e.g., TGF-β-Smad, PI3K-Akt-mTOR, p38 MAPK, NRF2/HO-1) across muscle cells, immune cells, vascular endothelia, and intracellular organelles (e.g., mitochondria, endoplasmic reticulum, Golgi apparatus) [3-8]. Immune cells (e.g., mast cells, neutrophils, monocytes, macrophages, lymphocytes, eosinophils) mediate these processes through receiving upstream signals and downstream pathway activation [3]. Critically, immune cells influence the development of muscle satellite cells, myoblasts, and myotubes by secreting cytokines or antibodies, thereby regulating skeletal myositis outcomes. Skeletal myositis comprises three categories: exogenous-induced skeletal myositis, dystrophic myopathies, and idiopathic inflammatory myopathies [9-11].

In exogenous-induced skeletal myositis, mild damage triggers compensatory regeneration, whereas severe injury promotes chronicity and organ failure (e.g., acute kidney injury) [12]. Irreversible chronic disorders (e.g., dystrophic myopathies, idiopathic inflammatory myopathies) may arise from aberrant signaling (gene mutations, protein misfolding, autoimmune antibody overexpression) that disrupt tissue repair [10,13]. This review analyzes the inflammatory mechanisms across all three myositis categories to elucidate immune cell roles, providing a foundation for mechanistic research and clinical therapeutics.

## 2. Immune cell regulation of exogenous-induced skeletal myositis

Exogenous-induced skeletal myositis primarily includes traumatic acute skeletal myositis, exercise-induced acute skeletal myositis, ischemic acute skeletal myositis and drug-related skeletal myositis.

### 2.1 Traumatic acute skeletal myositis (Fig. 1)

Traumatic acute skeletal myositis is primarily associated with physical injuries, impacts, ligament tears. Following injury, compromised myofiber membranes release cellular contents that activate the complement system, elevating blood levels of C3a and C4a, resulting in neutrophil and macrophage infiltration [14]. Simultaneously, resident skeletal muscle neutrophils and mast cells rapidly activate, releasing abundant pro-inflammatory cytokines (e.g., TNF-α, IL-1, IFN-γ) that recruit additional peripheral immune cells to the injured site [14,15]. These cytokines trigger rapid neutrophil influx within 2 hours, peaking at 6-24 hours [16]. Neutrophils produce IL-1 and IL-8 to induce monocyte migration. Furthermore, CCR2 (C-C chemokine receptor type 2) interacting with CCL2 (C-C motif chemokine ligand 2) drives Ly6C^hi^ monocyte differentiation into pro-inflammatory M1 phenotype, which secretes cytokines (e.g., IL-6, IL-1β, TNF-α) and highly expresses inducible nitric oxide synthase (iNOS), generating reactive free radical NO to induce cell apoptosis [17]. M1 macrophages also recruit infiltrating T cells [18].

**Figure 1.**
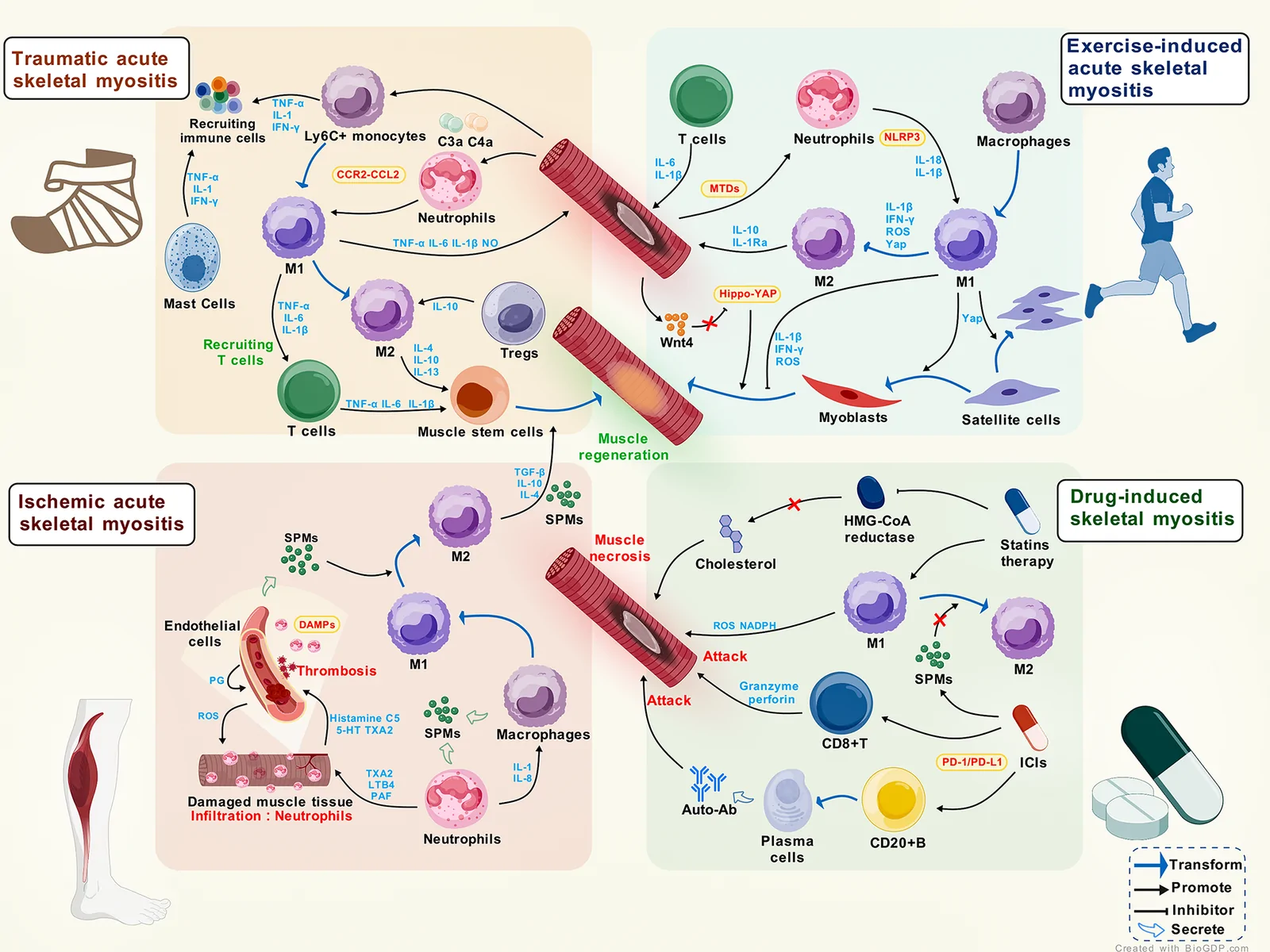
The Regulation of Immune Cells to exogenous-induced skeletal myositis.

Fu X et al. [19] revealed direct T cell-muscle stem cell (MuSC) interaction, where T cell-derived cytokines (IL-1α, IL-13, IFN-γ) sustain MuSCs activity, creating a microenvironment conducive to proliferation. As MuSCs expansion peak, macrophages switch from M1 to M2 phenotype, secreting anti-inflammatory cytokines (e.g., IL-4, IL-10, IL-13) to suppress local inflammation and promote MuSCs differentiation into myotubes [20]. Additionally, regulatory T cells (Tregs) secrete IL-10 to drive M1-to-M2 transformation.

Except for the clarification above, a clinical trial reveals that in elderly traumatic myositis, fibro-adipogenic progenitors (FAPs)-derived C3 recruits pro-inflammatory macrophages to clear apoptotic muscle cells while also enhancing FAPs’ phagocytosis of necrotic debris, highlighting its dual role in myofiber repair [21]. Therefore, timely macrophage phenotypic switching and regulation of C3 are crucial for muscle regeneration. IL-4-, IL-10-, or C3-targeted immunotherapy should be explored for traumatic acute myositis to alleviate symptoms and enhance muscle repair.

### 2.2 Exercise-induced acute myositis (Fig. 1)

Strenuous exertion triggers exercise-induced acute myositis, distinct from typical inflammation pathways. Moderate inflammation supports muscle repair via mitochondrial stress regulation [22]. Mitochondrial damage-associated molecular patterns (MTDs) activate pathogen-associated molecular patterns (PAMPs), stimulating neutrophils through formyl peptide receptor-1 and TLR9 [22]. MTDs increase Ca²^+^ flux in polymorphonuclear leukocytes (PMNs) and mitogen-activated protein kinase (MAPK) phosphorylation, promoting migration and particle release [23]. However, excessive MTDs disrupt mitochondrial function, impairing recovery [24]. Cellular damage activates NOD-like receptor pyrin domain-containing protein 3 (NLRP3) inflammasomes via DAMPs, releasing IL-1β and IL-18 via caspase-1 [25].

In the M1 phase, macrophages secrete IL-1β, IFN-γ, ROS, and Yes-associated protein (YAP) [26], followed by IL-10 and IL-1Ra in the M2 phase [27]. Transient inflammation aids regeneration, but prolonged activation exacerbates mitochondrial stress [28]. Dysregulated IL-6/IL-1β exacerbates dysfunction and delays repair. Failure to shift to M2 may cause atrophy [29]. Thus, precise regulation of inflammatory timing is crucial for recovery.

In injured muscle, myofiber- and macrophage-derived YAP promotes pro-inflammatory cytokine production (IL-6, IL-1β, TNF-α) [30,31] and enhances IL-1β expression by stabilizing the NLRP3 inflammasome [32]. Conversely, the Hippo/YAP pathway regulates regeneration: Wnt4-RhoA/ROCK signaling normally suppresses YAP to maintain satellite cell quiescence. Post-injury, reduced Wnt4 activates YAP, driving satellite cell proliferation [33,34]. Thus, while excessive YAP sustains inflammation, its controlled activation is essential for regeneration, warranting further investigation into its spatiotemporal regulation for therapeutic targeting.

Emerging evidence suggests that omega-3 (ω-3) polyunsaturated fatty acids may mitigate exercise-induced myositis. Clinical trials indicate that ω-3 supplementation integrates into skeletal muscle cells, reducing levels of IL-6 and oxidative stress while improving muscle membrane integrity [35]. This helps prevent leakage of intracellular proteins (e.g., creatine kinase [CK], lactate dehydrogenase [LDH]) [35]. However, further research is needed to establish optimal dosages and treatment durations for ω-3 supplementation.

### 2.3 Ischemic acute skeletal myositis (Fig. 1)

Unlike traumatic or exercise-induced myositis, ischemic myositis results from ischemia-reperfusion injury, commonly occurring as a surgical complication [36]. Vascular damage triggers a ‘damage control’ response: Platelets bind exposed collagen, while injured muscle releases inflammatory mediators (serotonin, histamine, thromboxane A2 [TXA2], C5 complement) [37-39]. Prostaglandins/leukotrienes promote thrombus formation to limit bleeding, followed by immune cell infiltration and localized inflammation [40]. Neutrophils peak at 24-48 hours, secreting IL-1/IL-8 to recruit macrophages [41]. Prolonged ischemia exacerbates reperfusion injury via cytokine-mediated vascular and myocyte damage [42]. IL-6/TNF-α stimulate endothelial reactive oxygen species (ROS) production, impairing function and increasing neutrophil adhesion [43-45]. Damage-associated molecular patterns (DAMPs) from necrotic cells activate pattern recognition receptors (PRRs), inducing IL-1β, IL-6, TNF-α, IL-8, and MCP-1 cascades [46]. Neutrophil-derived TXA2 generates leukotriene B4 (LTB4) and platelet-activating factor (PAF), amplifying vascular leakage and multi-organ injury risks [47]. Resolution relies on specialized pro-resolving mediators (SPMs) from neutrophils, macrophages, and endothelia [48]. SPMs promote granulocyte apoptosis, M1-to-M2 transition, and satellite cell activation for vascular/muscle repair [49]. Further research on SPM-driven apoptosis may enhance inflammation resolution and regeneration strategies.

### 2.4 Drug-induced skeletal myositis (Fig. 1)

Distinct from the preceding physical or metabolic insults, drug-induced skeletal myositis includes statin-induced and immune checkpoint inhibitor (ICI)-induced myopathies [50]. It disrupts myofiber necrosis/regeneration balance, leading to weakness and degeneration [51,52].

ICIs, like pembrolizumab, block programmed cell death 1/programmed cell death ligand 1 (PD-1/PD-L1) signaling, potentially triggering necrotizing myositis in melanoma therapy [53]. PD-1 normally suppresses self-reactive T cells [54,55], and its inhibition promotes cytotoxic CD8^+^ T-cell infiltration (granzyme/perforin-mediated damage) and autoantibody production (released by CD20^+^ B cells) due to CD25^+^ T cell deficiency (reducing SPMs, e.g., IL-4, IL-10, IL-13, TGF-β) [53,56,57]. Persistent PD-1/PD-L1 blockade impairs pro-inflammatory cell clearance and phenotypic switching, thereby exacerbating autoantibody-mediated injury.

Statin therapy, while beneficial, may cause myalgia or myositis, often with proximal weakness [58]. Anti-3-hydroxy-3-methylglutaryl-coenzyme A (HMG-CoA) reductase antibodies and elevated CK levels are common [59]. Statins reduce cholesterol synthesis, destabilizing muscle membranes and impairing function [60-62]. Intracellular calcium dysregulation and coenzyme Q10 (CoQ10) depletion exacerbate oxidative stress (via nicotinamide adenine dinucleotide phosphate [NADPH] oxidase-driven ROS), worsening sarcolemmal damage [63,64]. In-depth exploration of CD25^+^ T cells, autoantibodies, HMG-CoA, and CoQ10 mechanisms is essential for developing future therapies targeting drug-induced pathology.

Currently, immunosuppressive agents, particularly glucocorticoids, are the first-line therapy for drug-induced acute myositis due to their potent anti-inflammatory effects [65]. However, clinical evidence suggests that intravenous immunoglobulin (IVIg) may serve as a viable alternative in refractory cases where conventional immunosuppression fails to achieve adequate symptom control [66]. Furthermore, the management of drug-induced myositis must account for potential drug-drug interactions, as certain combinations can worsen myotoxicity or exacerbate disease severity. Therefore, a personalized treatment approach, incorporating thorough assessment of hepatic/renal function, concomitant medications, and individual risk factors, is essential to optimize therapeutic efficacy and minimize adverse effects.

Exogenous factors induce skeletal muscle damage, with sequential immune cell interactions playing crucial roles throughout injury stages. Notably, traumatic, exercise-induced, and ischemic acute skeletal myositis are primarily regulated by cellular immunity, whereas drug-induced skeletal myositis is governed by humoral immunity with cellular immunity playing a secondary role.

## 3. Dystrophic Myopathies

Dystrophic myopathies encompass heterogeneous disorders defined by dystrophic biopsy features [67]. These conditions commonly manifest as muscle weakness and atrophy and include progressive myodystrophies (e.g., Duchenne Muscular Dystrophy, Becker Muscular Dystrophy Facioscapulohumeral Muscular Dystrophy) and malnutrition-related muscle atrophy, the latter primarily associated with severe Myasthenia Gravis.

### 3.1 Duchenne Muscular Dystrophy (Fig. 2)

Duchenne Muscular Dystrophy (DMD), an X-linked recessive disorder, arises from out-of-frame mutations in the DMD gene causing chronic degeneration, weakness, and skeletal deformity [68,69]. Dystrophin deficiency impairs cytoskeleton-extracellular matrix linkage, leading to sarcolemmal fragility under mechanical stress, increased calcium permeability, and subsequent myocyte dysfunction/death [70]. Additionally, stretch-induced contractions generate reactive ROS, activating non-receptor tyrosine kinases, inducing stretch-activated channels (SACs) opening, and promoting calcium influx-further disrupting cellular integrity.

Damaged myofibers release DAMPs, activating innate immunity. Mast cells/neutrophils recruit M1 macrophages and CD4^+^ T/CD8^+^ T cells that secrete TNF-α, IL-6, and IFN-γ, driving damage and fibrosis. The inflammatory milieu decreases IL-10, IL-4, and amphiregulin (Areg), inhibiting M2 macrophage and Treg proliferation, and accelerating muscle necrosis [71,72]. However, in DMD, M1 macrophages exacerbate damage through pro-inflammatory mediators, while M2 macrophages sustain FAP survival. Critically, this M1/M2 imbalance-coupled with Treg impairment that disrupts M1-to-M2 transition, perpetuates inflammation and fibro-adipogenic replacement [73,74]. Therapeutic strategies should therefore modulate (not suppress) inflammation to restore immune/FAP balance [70].

MuSCs constitute a heterogeneous population essential for repair. Myofiber repair occurs through asymmetric MuSC division and interactions with dystrophin-associated protein complex (DAPC). Notably, research shows that abnormal polarity proteins significantly reduce asymmetric MuSC division in *mdx* mice, diminishing myogenic progenitor numbers. Moreover, *mdx* MuSCs exhibit impaired differentiation capacity and initiate fibrotic processes, suggesting MuSC dysfunction in DMD contributes to reduce muscle regeneration and increase fibrosis [70].

Current DMD treatment relies on glucocorticoids, delaying disease progression by 2-5 years, improving pulmonary function, and reducing scoliosis/cardiomyopathy risks [75]. Exon-skipping therapies (e.g., eteplirsen) show limited functional benefits for specific mutations. Emerging agents (e.g., TGF-β inhibitors, myostatin blockers) demonstrate partial effects—slowing fibrosis or increasing muscle mass—without consistent functional gains [76]. Optimal management combines glucocorticoids with genotype-tailored approaches, but future therapies require enhanced efficacy and mechanistic insights.

In contrast to DMD, Becker Muscular Dystrophy (BMD), caused by in-frame DMD mutations [69], presents milder/variable symptoms. Heier CR et al. [77] generated a BMD mouse model via CRISPR-mediated exon 45-47 deletion. Compared to DMD, BMD expresses truncated dystrophin isoforms, resulting in attenuated pathology, highlighting this model’s utility for therapeutic exploration.

**Figure 2.**
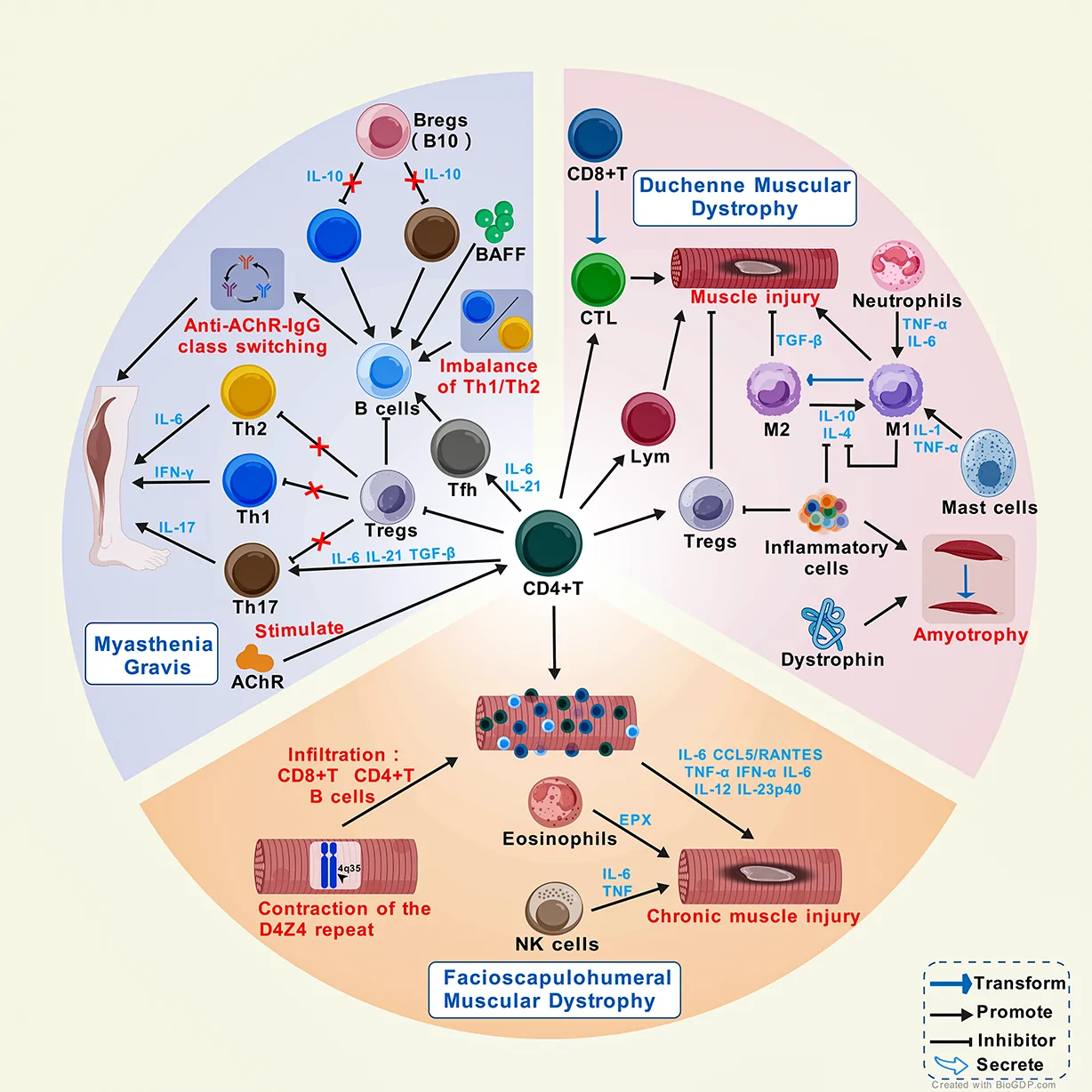
The Regulation of Immune Cells to DM.

### 3.2 Facioscapulohumeral Muscular Dystrophy (Fig. 2)

Distinct from DMD/BMD, Facioscapulohumeral Muscular Dystrophy (FSHD) progressively affects all ages/genders (prevalence: 1:8,300-1:15,000) [78,79]. FSHD1 (95% cases) involves contraction of D4Z4 macrosatellite repeats (<10 repeat units) on 4q35; FSHD2 stems from mutations in chromatin modifiers (e.g., structural maintenance of chromosomes flexible hinge domain containing 1, SMCHD1) [80]. Both induce D4Z4 chromatin relaxation, permitting toxic double homeobox protein 4 (*DUX4*) expression [79]. Muscle immune infiltration correlates with elevated serum CK. Histological analyses reveal abundant B cells and T cells, particularly endomysial CD8^+^ T cells and perivascular CD4^+^ T cells. These inflammatory cell characteristics increased serum levels of cytokines and chemokines (e.g., C-C motif chemokine ligand 5 [CCL5/RANTES], TNF-α, IFN-α, IL-6, IL-12, and IL-23p40) [79,81].

This immune involvement suggests that reducing T-cell infiltration (especially CD8^+^ T) may improve prognosis. Notably, Greco A et al. [79] reported elevated serum IL-6 in FSHD patients versus controls, with NK cells producing excess IL-6/TNF upon TLR stimulation. As a contraction-induced myokine, IL-6 directly contributes to weakness and atrophy [79], making it a clinically valuable biomarker. Further implicating immune dysregulation, Nunes AM et al. [78] observed significant increases in gastrocnemius eosinophil peroxidase (EPX) in chronic FSHD-like mice. Persistent *DUX4* expressions induce eotaxin and EPX, recruiting eosinophils [78]. Given eosinophils’ pro-fibrotic role in DMD (via TGF-β release or collagenase suppression), similar mechanisms likely operate in FSHD. EPX’s pathological contribution and eosinophil chemotaxis require further study.

Aguirre AS et al. [82] demonstrated that physical therapy, particularly low-to-moderate aerobic exercise, preserves muscle function and quality of life, albeit modestly (Standardized Mean Difference [SMD]=0.35; 95%CI: 0.12-0.58). While *DUX4*-targeting approaches (e.g., antisense oligonucleotides [ASOs], RNA interference [RNAi]) show preclinical potential, clinical evidence remains limited to small studies. Meta-analysis confirms available treatments—including drugs, rehab, and gene therapy—slow but do not halt progression. Advancing disease-modifying therapies require large randomized controlled trials, enhanced *DUX4* targeting, and multidisciplinary collaboration.

### 3.3 Myasthenia Gravis (Fig. 2)

While primarily a disorder of neuromuscular transmission, Myasthenia Gravis (MG) is fundamentally a B-cell-mediated autoimmune disorder causing weakness and fatigue [83]. Two main subtypes exist: acetylcholine receptor (AChR)-MG (IgG1-/IgG2-/IgG3-driven) and muscle-specific kinase (MuSK)-MG, both targeting neuromuscular junction proteins to impair transmission [84]. In AChR-MG, thymic abnormalities and T-cell dysregulation drive pathogenesis. AChR-specific CD4^+^ T cells secrete IFN-γ/IL-17, with Th17 activity correlating with disease severity [85,86]. Although Treg numbers remain normal, their impaired function elevates pro-inflammatory cytokines (IL-6, IL-17, IFN-γ) [87]. Expanded CD4^+^CXCR5^+^PD-1^+^ T follicular helper cells (Tfhs) promote germinal center (GC) formation via IL-21, enhancing autoantibody production [88].

Moreover, impaired central B-cell tolerance in AChR/MuSK-MG, potentially resulting from elevated IL-17, reduces B-cell migration within GC but enhances Tfh-B cell interactions [88,89]. Tfh cells facilitate positive selection of B cells to produce high-affinity autoantibodies. Consequently, targeting Tfh generation or their cytokine signals (IL-21, IL-17) may impede pathogenic autoantibody production. Another key regulatory deficit involves regulatory B10 (IL-10-producing B) cells, suppressing antigen-specific inflammation [90]. Guptill JT et al. [90] reported decreased B10 cells in MuSK-MG, explaining enhanced Th1/Th17 responses and loss of immune tolerance, ultimately generating autoreactive B cells. Furthermore, elevated B cell activating factor (BAFF) levels support the survival of these autoreactive B cells.

As for therapy, Zhong H et al. [91] analyzed MG treatments, finding corticosteroids offer fast relief but risk long-term side effects. Azathioprine reduces relapses (Odds Ratio [OR]≈0.45) but works slowly, while rituximab (RTX) benefits refractory MuSK positive patients. Complement inhibitors improve Quantitative Myasthenia Gravis (QMG) score (OR=2.5) in AChR positive cases. For thymectomy, non-thymoma patients show possible long-term benefit, though evidence is mixed [92]. Thymoma patients require surgery plus immunotherapy. Treatment choices must balance efficacy, safety, and cost, with biologics and surgery supplementing core immunosuppressants.

These dystrophic disorders share muscle weakness/atrophy and complex pathomechanisms. In DMD, ROS generation and calcium dysregulation activate immune cells, driving muscle damage and fibrosis. In FSHD, immune infiltration and cytokine elevation sustain chronic inflammation and degeneration. Though etiologically distinct, MG, DMD and FSHD all involve dysregulated immune responses and cytokine signaling. Critically, immune system imbalance, mediated by T/B cells and cytokines, induces muscle damage and dysfunction. Restoring immune equilibrium is thus mitigating progression and improving outcomes.

## 4. Idiopathic Inflammatory Myopathies

Idiopathic Inflammatory Myopathies (IIMs) are a group of rare chronic autoimmune diseases characterized by muscle inflammation. Common symptoms of IIMs include muscle weakness, reduced muscle endurance, and myalgia. Additionally, extramuscular manifestations, such as rash, arthritis, and interstitial lung disease (ILD), are common. IIMs encompass five subtypes: polymyositis, inclusion body myositis, dermatomyositis, antisynthetase syndrome and immune-mediated necrotizing myopathy.

### 4.1 Polymyositis (Fig. 3)

Polymyositis (PM) is a subtype of IIMs characterized by progressive, symmetric proximal muscle weakness. Histopathology reveals inflammatory infiltrates dominated by macrophages and CD8^+^ cytotoxic T lymphocytes (CTLs), with non-necrotic myofibers expressing MHC-I [93,94]. This immunological microenvironment facilitates a specific pathogenic cascade: Antigen-specific CTLs secrete IFN-γ, TNF, and high-mobility group box 1 (HMGB1), inducing MHC-I upregulation on myofibers. Furthermore, the upregulation of co-stimulatory molecules (CD80 [B7-1]) and inducible co-stimulator ligand (ICOSL), along with their corresponding ligands (e.g., CD28, cytotoxic T-lymphocyte-associated protein 4 [CTLA-4], and ICOS), together with intercellular adhesion molecule 1 (ICAM-1) or lymphocyte function-associated antigen 1 (LFA-1) molecules, stabilizes synaptic interactions between CD8^+^ T cells and MHC-I^+^ myofibers [94,95]. This indicates myofibers can function as antigen-presenting cells.

The consequences of this aberrant immune activation are threefold: Firstly, autoreactive CD8^+^ T cell clones expand and release perforin and granzyme B, mediating myofiber necrosis. Research has demonstrated that CD8^+^ CTLs contribute to myofiber injury through cytotoxic effects [95]. Secondly, CD28-deficient T cells, a group of pro-inflammatory, terminally differentiated, and long-lived cells, modulate muscle T-cell infiltration. Their absence is associated with immunosuppression resistance and adverse manifestations [96]. Thirdly, B-cell responses in PM muscle tissue drive B-cell and plasma cell infiltration, indicating chronic antigen-driven humoral immunity. Significant upregulation of clonally derived immunoglobulin transcripts and BAFF in biopsies suggest local plasma cell maturation, perpetuating inflammation [97]. Collectively, these findings indicate in PM patients, targeting pathogenic CD8^+^ T cells, B cells, plasma cells within the muscle, or antagonizing the cytokines they release, can help delay disease progression.

Recently, clinical evidence demonstrated that B cell depletion therapy using rituximab achieves approximately 69% efficacy in steroid-refractory cases, validating autoreactive B cell elimination as a viable therapeutic approach [98]. Emerging strategies focus on modulating T cell responses, including abatacept (CTLA-4-Ig) fusion proteins that interrupt CD28-mediated co-stimulation, showing promise in clinical trials for reducing T cell-mediated myocyte injury [99].

**Figure 3.**
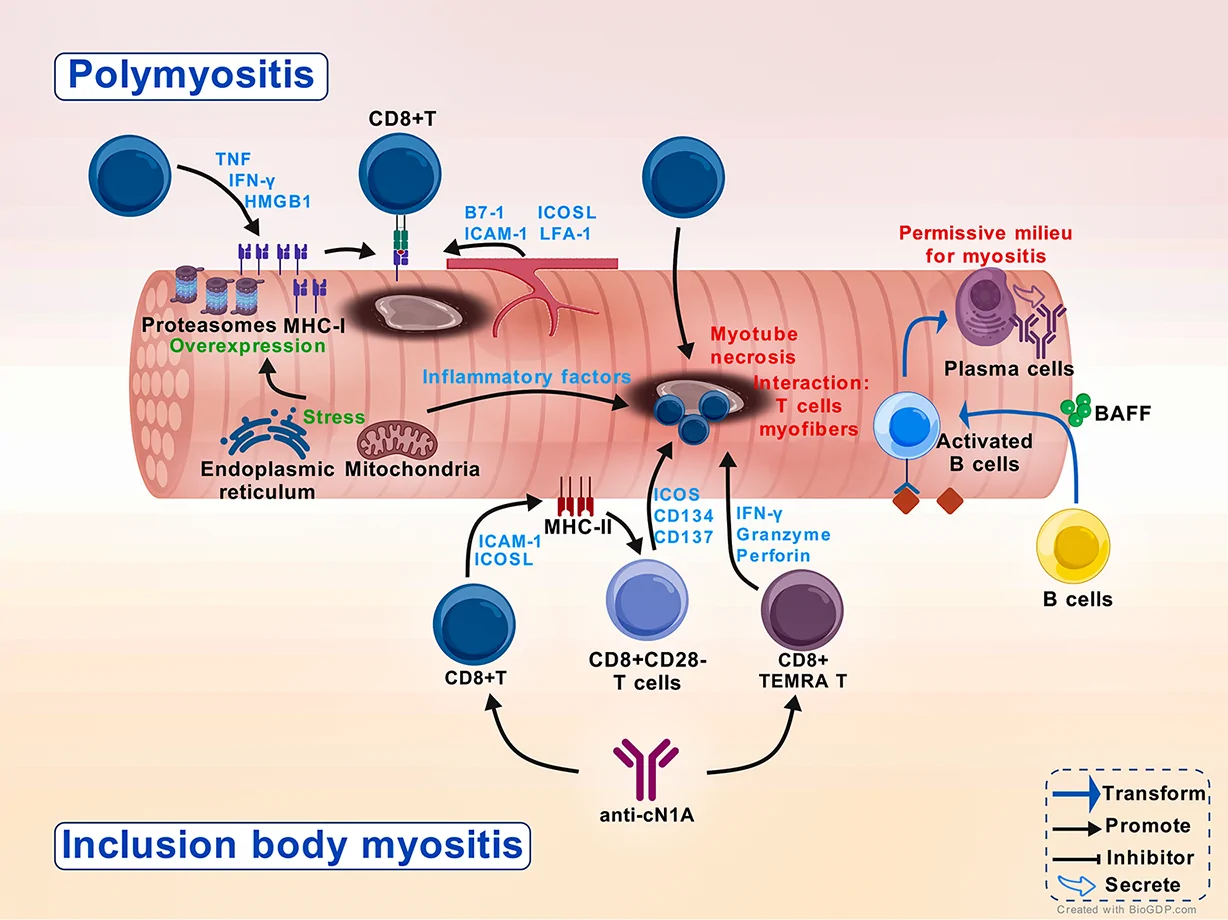
The Regulation of Immune Cells to PM and IBM.

### 4.2 Inclusion body myositis (Fig. 3)

Inclusion body myositis (IBM), the most common acquired myopathy in adults over 50 years, leads to progressive weakness and dysphagia [100]. Distinct from polymyositis, IBM features T cells, macrophages, and plasma cells infiltrating non-necrotic myofibers, releasing IFN-γ, chemokines, and granzymes [100,101]. CD8^+^ CTLs target antigen-presenting myofibers, activating CD4^+^ responses via MHC-II, ICAM-1, and ICOSL upregulation [102]. Notably, CD8^+^CD28^-^ T cells expressing ICOS, CD134, CD137 mediate direct myofiber interactions [103].

Anti-cytosolic 5'-nucleotidase 1A (*cN1A*) autoantibodies activate CD8^+^ terminally differentiated effector memory (TEMRA) T cells, triggering IFN-γ/perforin-mediated damage [104]. Concurrently, immune proteasomes colocalize with MHC-I^+^ fibers, inducing mitochondrial dysfunction, endoplasmic reticulum (ER) stress and oxidative injury [105]. These processes drive vacuolization and denervation. Therapeutically, targeting CD8^+^ T cells or IFN-γ may mitigate dysfunction.

Therapeutic development has centered on interrupting the IFN-γ pathway and targeting cytotoxic T cells [106]. Experimental models showed anti-CD3 monoclonal antibody (OKT3) reduced T-cell infiltration and MHC-I overexpression in IBM xenografts, suggesting promising intervention strategies [107]. While traditionally refractory to immunosuppression, novel agents targeting senescent T cells or IFN-γ signaling, combined with protein homeostasis modulators, may offer disease-modifying potential.

**Figure 4.**
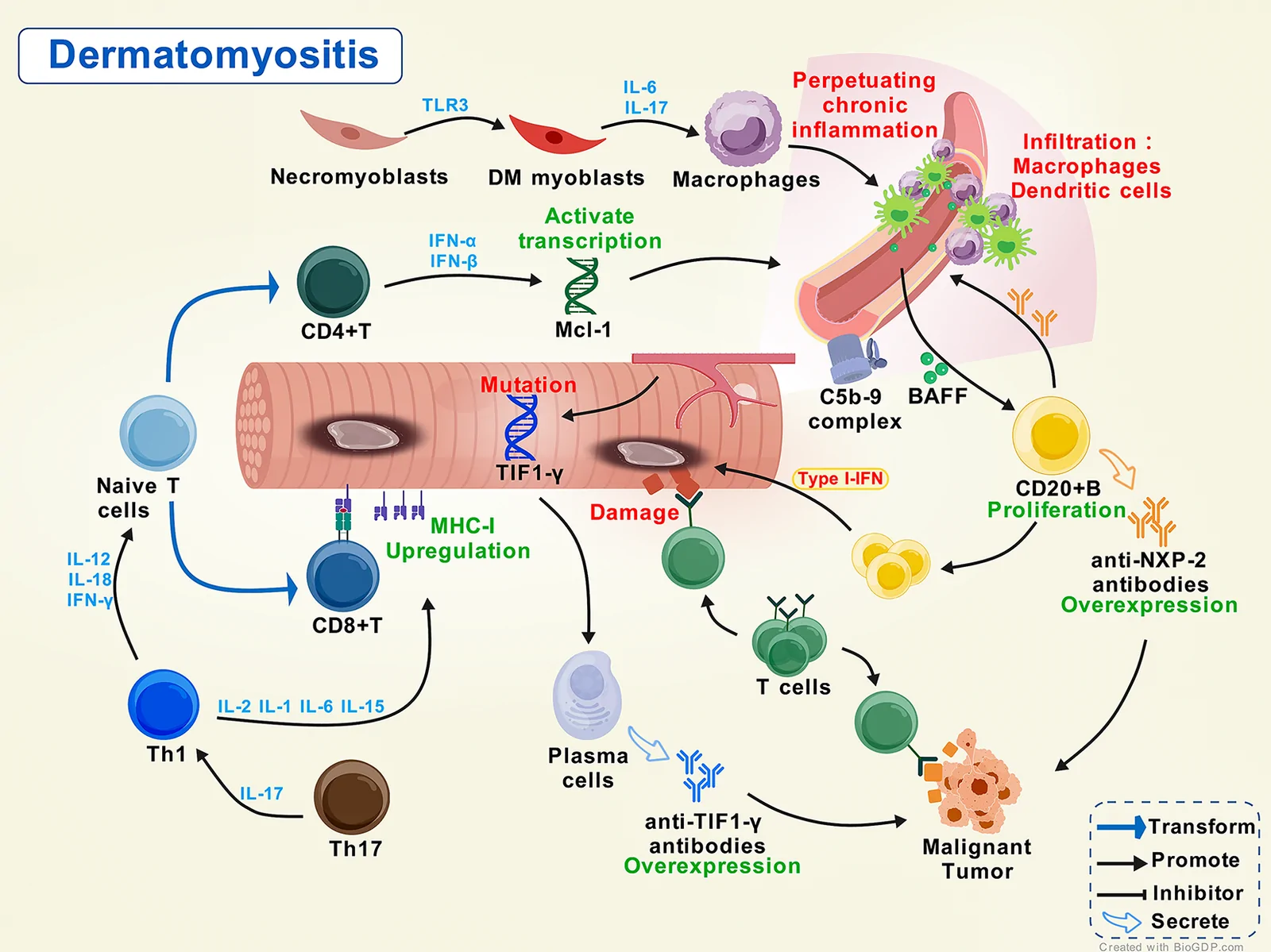
The Regulation of Immune Cells to DM.

### 4.3 Dermatomyositis (Fig. 4)

Transitioning from T-cell pathology to IBM, dermatomyositis (DM) emerges as the most common IIM, marked by proximal muscle weakness and pathognomonic skin lesions like Gottron papules and heliotrope rash. Its pathogenesis involves humoral autoimmunity, complement dysfunction, and interferon overexpression [108].

Histopathology shows perivascular and perifascicular infiltrates of CD8^+^/CD4^+^ T cells, macrophages, and dendritic cells [109]. A dominant interferon signature is seen, with CD20^+^ B cells expressing type I-interferon (IFN)-inducible genes (MxA) and endothelial tubuloreticular inclusions marking type I-IFN activation [110,111]. In melanoma differentiation-associated protein 5-positive (MDA5^+^) DM, elevated BAFF and anti-tripartite motif containing 21 (TRIM21)/splicing factor proline and glutamine rich (SFPQ) autoantibodies indicate B-cell dysfunction [112]. ScRNA-seq analyses reveal expanded antibody-secreting cells and activated CD8^+^ T-cell responses in affected tissues [112].

T cells are pivotal in DM pathogenesis [113]. Dysregulated CD4^+^ T cells drive IFN-α-/IFN-β-induced anti-apoptotic myeloid cell leukemia-1 (Mcl-1) expression [114], while Th1-derived IFN-γ and IL-12/IL-18 [115]. Necrotic myoblasts activate Toll-like receptor 3 (TLR3)-mediated IL-6/IL-17 [116], and CD28^-^ T cells amplify inflammation via IL-17 from Th17 cells to trigger the release of pro-inflammatory Th1 cytokines (e.g., IL-2, IL-1, IL-6, IL-15) and MHC upregulation on myofibers [117]. Microvascular injury is further evidenced by membrane attack complex (C5b-9) deposition.

DM’s humoral response shows distinct clinical-autoantibody correlations. Anti-transcription intermediary factor 1-gamma (TIF1-γ) and anti-nuclear matrix protein 2 (NXP-2) antibodies are strongly associated with cancer-associated myositis (60-80% malignancy-linked) [118]. Normally regulating Smad signaling, tumor-altered TIF1-γ may trigger autoimmunity via molecular mimicry [119-121], driving CD8^+^ T-cell-mediated myositis [120]. Anti-NXP-2 exhibits age-specific effects: causing juvenile calcification while potentially promoting adult tumors via p53 suppression [122].

Meta-analytical data indicate tacrolimus-based triple therapy outperforms cyclosporine regimens in reducing mortality and oxygen dependence [123]. While JAK inhibitor-containing quadruple therapy expands options, its elevated infection risk necessitates careful risk-benefit assessment [124].

In summary, DM treatment targets three key pathways: Firstly, JAK inhibitors show promise for anti-TIF1-γ^+^ DM by modulating genetic factors [125]. Secondly, CTLA-4-Ig blocks T-cell activation via CD80/CD86-CD28 interaction blockade [126]. Thirdly, B-cell depletion may help counter pathogenic antibodies, though specific inhibitors remain lacking. These targeted approaches reflect advances in understanding DM’s complex immunopathology, with combination strategies potentially improving outcomes for refractory cases.

### 4.4 Anti-synthetase syndrome (Fig. 5)

In contrast to DM’s prominent cutaneous and malignant associations, anti-synthetase syndrome (ASS) presents a distinct clinical profile centered on pulmonary involvement. Defined by anti-aminoacyl-tRNA synthetase (ARS) antibodies, ASS typically manifests with ILD, myositis, arthritis, fever and Raynaud's phenomenon [127], with ILD representing the most frequent and clinically significant manifestation [128].

The ten identified ARS antibodies, including anti-histidyl tRNA synthetase antibody (Jo-1) and anti-threonyl-tRNA synthetase antibody (PL-7), initiate pathogenesis through unique mechanisms. Beyond their role in protein biosynthesis, ARS enzymes influence immune regulation when forming antigen-antibody complexes. Notably, anti-Jo-1 antibodies demonstrate particularly strong musculoskeletal associations, with over 90% of positive patients developing severe proximal weakness and myalgia [129]. The autoimmune cascade begins when NK cell-derived granzyme B cleaves histidyl-tRNA synthetase (HisRS) into antigenic fragments [130,131], creating autoantigens that promote T-cell proliferation via CCR5-mediated chemotaxis and CD40L upregulation, leading to IFN-γ, IL-2 and IL-17 production [131].

Muscle pathology in ASS reveals highly organized immune architecture. CD68^+^CD169^+^ macrophages dominate perimysial regions, facilitating persistent antigen presentation [131], while ectopic germinal centers form between muscle bundles through coordinated B-cell and follicular dendritic cell interactions [132]. ScRNA-seq analyses demonstrate how CXCL12/CXCL13 gradients sustain this microenvironment, enabling continuous high-affinity antibody production. This lymphoid organization creates a self-perpetuating cycle where B-cell-derived inflammatory mediators (IL-6, lymphotoxin-α/β [LT-α/β]) synergize with IFN-γ to upregulate myofiber MHC expression, expanding the antigenic targets [132].

In ASS myositis, immune cell infiltration exhibits distinct spatial organization: T cells predominate in perimysial regions, while CD20^+^ B cells and CD138^+^ plasma cells cluster at perimysial-endomysial junctions. Tfh cells within interstitial tissues secrete IL-21 and CD40L, establishing a T-B cell amplification loop that sustains inflammation through CXCL12-/CXCL13-mediated recruitment [132]. This cascade drives CD8^+^ T cell activation and subsequent B cell-mediated antibody production [131]. Therapeutic interventions targeting these pathways show clinical efficacy: RTX (anti-CD20) reduces muscle enzymes and improves pulmonary function, while abatacept disrupts CD28-CD80/86 co-stimulation, ameliorating ASS-ILD manifestations in controlled clinical trials [133].

### 4.5 Immune-mediated necrotizing myopathy (Fig. 5)

Distinct from other IIM subtypes, immune-mediated necrotizing myopathy (IMNM) presents a unique antibody-driven pathogenesis. IMNM involves specific autoantibodies that mediate myofiber injury through complement-dependent mechanisms, with pathological hallmarks including macrophage-mediated phagocytosis of necrotic myofibers [134]. The disease is subclassified by its characteristic autoantibodies, with most cases associated with either anti-signal recognition particle (anti-SRP) and anti-HMG-CoA reductase (anti-HMGCR) antibodies, along with a smaller seronegative group [135]. Experimental evidence strongly supports the direct pathogenicity of these autoantibodies. Plasma from anti-SRP^+^ or anti-HMGCR^+^ IMNM patients significantly reduce cultured myotube area while altering cytokine profiles, which decrease IL-4 and IL-13 secretion and increasing pro-inflammatory mediators (e.g., IL-6, TNF, and ROS) [136]. These cellular changes correlate with molecular markers of atrophy, including upregulated TRIM63/MuRF1 and MAFbx/atrogin-1 mRNA, demonstrating how autoantibodies impair muscle survival and regeneration [136].

**Figure 5.**
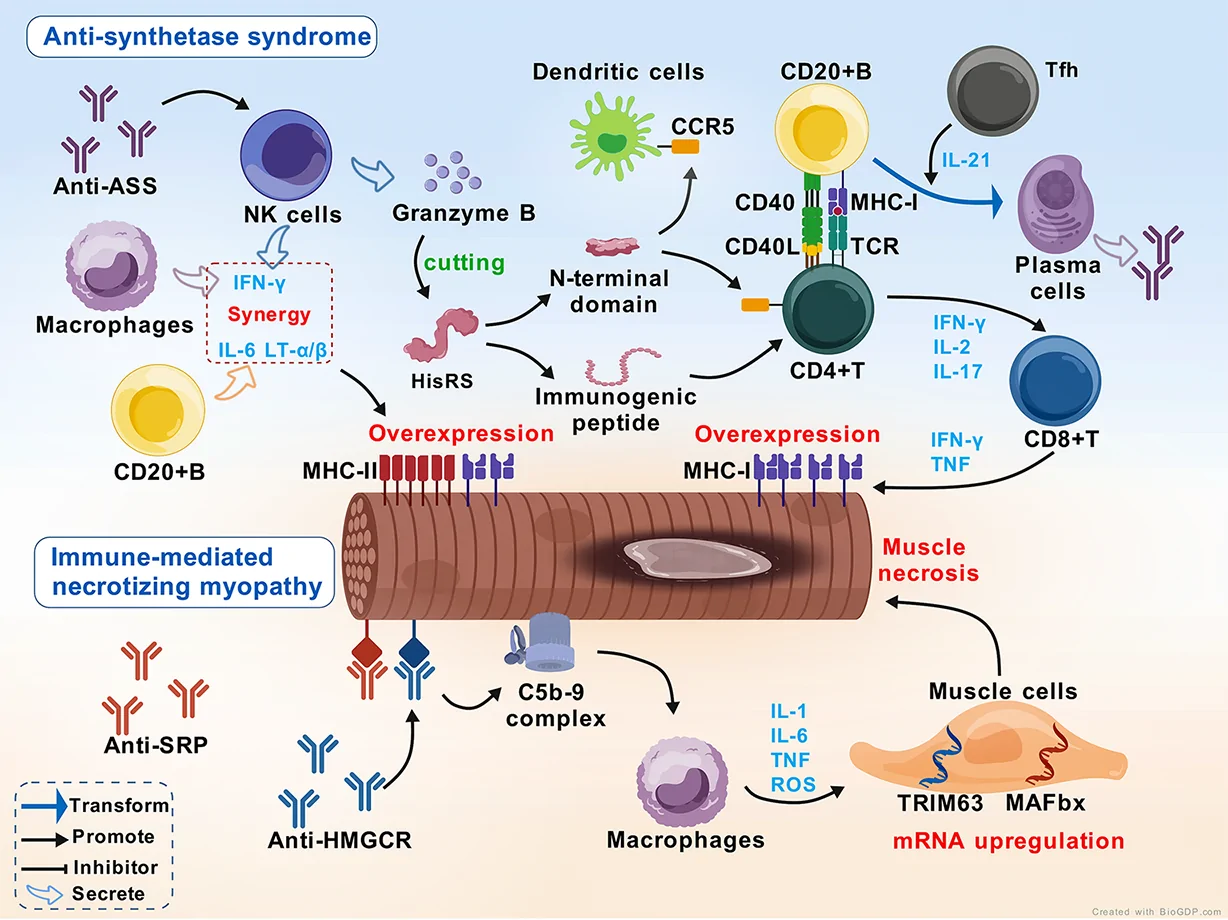
The Regulation of Immune Cells to ASS and IMNM.

The complement system plays a central role in translating autoantibody binding into tissue damage. Autoantibody binding activates classical complement cascade, leading to membrane attack complex formation and subsequent necrosis. This initiates a destructive cycle: Macrophage recruitment results in muscle phagocytosis and pro-inflammatory cytokine (e.g., IL-1, IL-6, TNF) release [137]. Compelling animal studies validate this mechanism, showing that transferred patient autoantibodies induce muscle dysfunction in mice, with pathogenicity being complement-dependent-diminished in C3-deficient mice but enhanced with human complement supplementation [138]. These pathogenic insights directly inform therapeutic strategies. The strong evidence for antibody-mediated pathology supports targeted approaches including plasmapheresis and novel complement inhibitors. Notably, animal models of active immunization that break self-tolerance successfully replicate IMNM features, further validating the autoimmune basis and potential treatment approaches [138].

Current management emphasizes pathogenic autoantibody neutralization. High-dose IVIg and plasmapheresis effectively mitigate symptoms by antibody removal and complement inhibition, with the latter particularly beneficial for acute severe cases [139]. Although C5 inhibitor trials showed limited efficacy [140], earlier complement cascade intervention or direct antibody suppression may prove more effective. Treatment algorithms typically combine aggressive immunosuppression with IVIG, reserving rituximab or antibody removal therapies for refractory cases [141].

In summary, while IIMs subtypes vary clinically and pathologically, muscle alterations primarily stem from aberrant immunity, including immune cell infiltration, cytokine release, and immune complex formation. Specifically, CD8^+^ CTLs contribute to excessive MHC-I expression on myofibers by secreting pro-inflammatory cytokines, which in turn initiate inflammation and tissue damage. Furthermore, activation of CD4^+^ Th cells and B cells further modulates and intensifies immune responses. The dysregulation of these inflammatory cells and cytokines drives muscle inflammation, degeneration, and atrophy, ultimately compromising function.

## 5. Discussion

Immune cells critically shape prognosis across myositis subtypes, with distinct cytokine profiles and signaling pathways guiding therapeutic development [142-144]. Notably, while exogenous-induced, dystrophic, and IIMs all feature immune infiltration and cytokine dysregulation, their pathophysiology diverges significantly. Exogenous triggers (e.g., trauma, drugs) induce acute inflammation dominated by neutrophils, macrophages (M1/M2), and T cells—a process typically reversible but prone to chronicity if damage persists. In chronic cases, sustained neutrophil/macrophage-driven necrosis prevails. Importantly, M2 macrophages aid resolution via anti-inflammatory cytokine release and apoptotic cell clearance, facilitating muscle regeneration [145,146]. Thus, acute therapies should prioritize optimizing the M1-to-M2 transition while modulating immune recruitment.

In contrast to exogenous triggers, dystrophic myopathy arises from genetic mutations and exhibits immune dysregulation with predominant pro-inflammatory responses that actively hinder muscle repair [147,148]. Meanwhile, IIMs are characterized by chronic autoimmune inflammation mediated by immune cell infiltration, cytokine release (e.g., IFN-γ, TNF-α, IL-6), and complement activation. Strikingly, while exogenous myositis features prominent neutrophil/macrophage infiltration, dystrophic myopathy shows minimal immune involvement, and IIMs rely more on cytokine-mediated damage.

Given these pathological distinctions, therapeutic strategies must be tailored to each subtype. For ischemic myositis, adipose-derived stem cell (ADSC) therapy combined with growth factors has emerged as a promising approach. For instance, Wang R et al. [149] developed methacrylated gelatin (GelMA) microspheres to co-deliver ADSCs and fibroblast growth factor 19 (FGF19), enabling targeted stem cell delivery and sustained growth factor release to enhance regeneration.

Beyond ischemia, drug-induced myositis (e.g., statin/ICI-induced) involves predominant humoral immunity, where persistent drug exposure triggers pathogenic autoantibody production via B-cell differentiation [56]. These autoantibodies disrupt PD-1/PD-L1 signaling, target muscle receptors, and promote membrane attack complex formation, collectively inducing myocyte necrosis [56-58]. Consequently, therapies for this subtype should focus on B-cell activation suppression and autoantibody neutralization. Similarly, for traumatic/exercise-induced myositis, combinatorial approaches (e.g., immunomodulator co-delivery systems) warrant exploration.

While glucocorticoids remain a cornerstone for dystrophic myopathy and IIMs, their limitations are increasingly apparent. In BMD, gene therapy advances (e.g., antisense oligonucleotides, adeno-associated virus [AAV] vector-mediated microdystrophin) face durability and immunogenicity challenges [150,151]. Similarly, while JAK inhibitors show promise in DM, the failure of alefacept in IBM underscores the disease’s complexity [151].

The advent of scRNA-seq is revolutionizing myositis research by decoding skeletal muscle heterogeneity. Nelke C et al. [152] identified senescent FAPs and inflammatory myonuclei (C3+/HLA-A+) in IBM, alongside expanded immune populations. Complementing this, Wang Y et al. [153] used scRNA-seq to reveal pathogenic macrophage activation in dystrophic mice, enabling novel biomarker discovery. Further demonstrating its versatility, Sinning J et al. [154] applied scRNA-seq to kidney aging, providing a methodological blueprint for skeletal muscle research—including cell subpopulation identification, senescence characterization, and cross-species validation. However, challenges like tissue variability and immune complexity persist. Future integration with mass cytometry could refine cell subtype characterization, accelerating therapeutic development and personalized approaches.

Emerging targeted therapies are now shifting paradigms beyond conventional treatments. For FSHD, novel agents like losmapimod and atomoxetine-oxybutynin combination 1020 (AOC-1020) directly inhibit toxic *DUX4* expression to slow degeneration [155,156]. Epigenetic editing therapies, such as EPI-321 (an AAVrh74 vector), aim to restore methylation in the D4Z4 region, silencing *DUX4* [157,158].

Other innovative strategies include the myostatin antibody (an anti-latent myostatin Fc-engineered antibody with increased affinity to FcγRIIb) and clustered regularly interspaced short palindromic repeats/CRISPR-associated 9 (CRISPR/Cas9)-based technologies [159]. Unlike BMD, where glucocorticoids are standard and cardiomyopathy risk exceeds 70%, FSHD rarely involves cardiac issues, allowing focused monitoring. Promising avenues include BCMA-targeted T-cell engagers, anti-FcRn agents [160], and macrophage-directed therapies to curb inflammation and boost regeneration [161]. Parallel advances in MG, such as C5 inhibitors (e.g., zilucoplan) and FcRn antagonists (e.g., rozanolixizumab), further highlight the potential of biologics [159,162].

Despite these advances, challenges remain short-lived efficacy, significant side effects, and disease heterogeneity impacting outcomes. To address these gaps, future research should prioritize multi-omics and artificial intelligence (AI)-driven mechanistic studies to dissect molecular differences and refine therapeutics. Key strategies include designing combination therapies (e.g., gene therapy with immunosuppression) and optimizing the local immune microenvironment (e.g., reducing fibrosis, modulating cell populations). Additionally, precision clinical trials incorporating biomarker screening will ensure homogeneous cohorts and improved sensitivity [163]. Continued exploration of emerging therapies—from BCMA-targeted agents to anti-FcRn treatments—will further expand the therapeutic arsenal for myopathies.

## 6. Conclusions

In summary, this review examines the regulatory role of immune cells in skeletal myositis, encompassing their impact on disease progression, repair, and regeneration across various diseases. We classify and analyze the underlying cellular and molecular biology exogenous-induced skeletal myositis, dystrophic myopathies and idiopathic inflammatory myopathies to enhance understanding of pathogenesis. The aim is to provide a theoretical foundation for further research into skeletal myositis and to inform clinical drug development.
